# Foodomics: Current and Future Perspectives in Food Analysis

**DOI:** 10.3390/foods11091238

**Published:** 2022-04-26

**Authors:** Yelko Rodríguez-Carrasco

**Affiliations:** Department of Preventive Medicine and Public Health, Food Science, Toxicology and Forensic Medicine, Faculty of Pharmacy, University of Valencia, Burjassot, 46100 Valencia, Spain; yelko.rodriguez@uv.es; Tel.: +34-963544228

Climate change, an increase in population, and the recent pandemic crisis triggered by SARS-CoV-2 have all contributed to a period of global problems. Due to increased food traffic and the number of prospective customers, it is vital to ensure food safety in terms of quality, traceability, and safety in this situation. Furthermore, environmental stressors such as meteorological disasters, rising global temperatures, and rising CO_2_ levels are on the rise and reducing crop yields. Moreover, there is an increasing interest in the diet–health dyad, as well as the link between certain chronic non-communicable diseases and diet. All of these new demands and problems are difficult to meet using traditional analytical chemistry in the food sciences.

Foodomics was conceptualized to address the issues of food science. It is characterized as a discipline that explores the fields of food and nutrition through the application of omics technologies. This discipline employs high-resolution techniques such as mass spectrometry, nuclear magnetic resonance, and next-generation sequencing, as well as a variety of mathematical and computer tools to handle and interpret data.

As a result, high-resolution analytical techniques are gradually being introduced into the food sciences, along with instruments that allow for the interpretation of the huge amounts of data generated. Because of their immense utility and development potential, considerable emphasis is currently made on the development of resolution power, analysis speed, affordability, and downsizing of these instruments due to the growth in food safety standards.

Food safety is a critical component of the food production and distribution process. Toxicological analyses allow for the qualitative and quantitative detection of substances and organisms that have the potential to affect people′s health (food hazards) or that can signal their presence directly or indirectly. Because of the vast quantity of information that can be collected from a single examination, foodomics allows for the adoption of approaches capable of detecting and evaluating new food hazards, as well as better efficiency in toxicological research. Foodomics can also help to understand how the production chain′s operations affect food, allowing for the development of optimization, assessment, and monitoring strategies. On the other hand, adulterations and fraud, both unintentional and intentional for financial benefit, are a serious problem that needs to be addressed. These actions cost the food business some billion euros and jeopardize public health because adulterated foods may contain ingredients that are capable of triggering diseases in customers who are ignorant of their presence. Hence, food fraud is also being investigated and controlled throughout omics technologies.

This Special Issue intends to contribute to the growth of this discipline in order to highlight its prospective uses and how it fits into the current scientific landscape in the area of food analysis.

Martínez-Alonso et al. (2022) [1] demonstrated that red beans are a rich source of bioactive substances such as flavanols and anthocyanidins, which offer antiradical activity and human health advantages, according to the chemical profile obtained from this study. In their work, red bean extract (diluted 1:8) demonstrated a significant increase in human liver cancer cell line (HepG2) proliferation after 24 h of exposure. Additionally, diluted red bean extract resulted in a reduction in reactive oxygen species (ROS) generation compared to the control at 120 min. The latter result was related to antioxidant activity, implying that the red bean extract could modulate oxidative stress in HepG2 cells.

Shi et al. (2022) [2] revealed the complimentary patterns of nutrient accumulation in distinct species and decoded the species-specific patterns of bioactive chemicals in three key staple crops and three fruits. Sweet corn had the most vitamins and amino acids of the three crops, but rice and wheat were vitamin and amino acid deficient. Mango was the most vitamin- and amino-acid-rich of the three fruits. Crops were high in fats compared to fruits. Overall, this research presented metabolomic evidence for a healthy diet, emphasizing the importance of macronutrients and micronutrients.

Lee et al. (2021) [3] used untargeted metabolite profiling methodologies to conduct a complete metabolite profiling of various soy fermented products and raw soybeans. It was reported in this research that variations in the metabolomes and volatolomes of soy products may be altered by changing processing steps, microbial successions, and fermentation time.

Keawkim et al. (2021) [4] highlighted that combined metabolomics and flavoromics could be utilized to identify the major metabolites and flavor components that vary in sacha inchi seeds during germination with the aim of establishing the optimum nutritional values and guide the improvement of pharmaceutical products or functional meals manufactured from sacha inchi seeds. In this research, it was reported that germinated sacha inchi seeds have higher levels of amino acids, organic acids, total phenolic compounds, and antioxidant activity than ungerminated seeds.

Uršulin-Trstenjak et al. (2021) [5] applied foodomics as a tool for the development of healthy food that is tailored to individual health issues and the prevention of food-related disorders in order to promote human longevity. Diet difficulties, which often lead to malnutrition, are common among the elderly in nursing facilities. To avoid nutritional danger, this study emphasizes the significance of examining and regularly monitoring the nutritional health of persons in elderly homes.

Rocchetti et al. (2021) [6] evaluated the potential of a metabolomics-based technique combined with retrospective high-resolution mass spectrometry (HRMS) screening to evaluate the mycotoxin profile of bulk milk samples. Overall, the HRMS technique putatively identified 46 mycotoxins. Zearalenol, mycophenolic acid, tentoxin, and apicidin were revealed to be among the most discriminant markers in this study. Although further confirmation studies were required, this work suggested potential carry-over and metabolization phenomena of mycotoxins in milk.

Cai et al. (2021) [7] reported that long-term 1-methylcyclopropene (1-MCP) treatment produces papaya ripening disorder, while appropriate 1-MCP treatment significantly prevents the ripening of papaya fruit. Their findings suggested that by targeting the ethylene and auxin signaling pathways, the miRNAs may play a significant role in regulating fruit ripening. Unsuitable 1-MCP therapy can cause fruit ripening disease by disrupting miRNA function.

The study of Kafantaris et al. (2021) [8] was the first to use a global transcriptome method to evaluate pine honey′s antibacterial effects and mechanism of action. Pine honey had a substantial effect on the transcriptomic profile of *Pseudomonas aeruginosa*. This study demonstrated that pine honey had a suppressive effect on *P. aeruginosa* genome expression, with more genes down-regulated than up-regulated. These findings could help to unravel the molecular pathways and biological processes involved in pine honey′s antibacterial action, which could aid in treating and control *P. aeruginosa* infection and pathogenicity.

Yu et al. (2021) [9] found that age had a substantial impact on meat quality parameters and meat exudate metabolome profiles. Metabolites linked to ATP synthesis and metabolism (creatine and hypoxanthine), antioxidation (GSSG and carnosine), and proteolysis (dipeptides and tripeptides) could be used as biomarkers to track aging times and detect changes in meat quality, such as increased lipid and protein oxidation, discoloration, and tenderness.

Based on the above-mentioned studies, the assurance of food quality and safety will be the foremost need in the future of food sciences. In the coming years, the worldwide circulation of food and related raw materials will expand, resulting in an increase in contamination. Furthermore, numerous processed items created with various ingredients from around the world will share storage rooms and production lines, making food safety incidents more difficult to regulate. As a result, assuring food safety, quality, and traceability will be considerably more difficult and critical than it is now. Foodomics is currently demonstrating its potential to respond to the demands of food science, even though it will take more time to develop, consolidate, and gain recognition. Based on the manuscripts listed in this Special Issue and on the others found in the literature, foodomics has a wide range of experimental methodologies and applications in the areas of food safety, quality control, authenticity, food process design, biotechnology, and nutrition. The existence of these current and future challenges, which are still far from being fully resolved, highlights the need to continue working on these issues in order to acquire a comprehensive perspective on the complicated situation in food and nutrition sciences.

## Data Availability

No new data were created or analyzed in this study. Data sharing is not applicable to this article.
